# Upper Gastrointestinal Endoscopy Findings in a Tertiary Centre in Pokhara: A Descriptive Cross-sectional Study

**DOI:** 10.31729/jnma.5927

**Published:** 2021-02-28

**Authors:** Durga Dhungana, Yukta Narayan Regmi

**Affiliations:** 1 Department of Internal Medicine, Gandaki Medical College Teaching Hospital and Research Center, Pokhara, Nepal

**Keywords:** *endoscopy*, *gastritis*, *indications*, *ulcer*

## Abstract

**Introduction:**

Endoscopic examination is one of the commonest procedures done in day-to-day practice in evaluating gastrointestinal problems. Esophagogastroduodenoscopy provides an excellent view of mucosal surfaces of the esophagus, stomach, and proximal duodenum. Upper gastrointestinal endoscopy is utilized for various diagnostic and therapeutic reasons. This study aimed to study the upper gastrointestinal endoscopy findings in a tertiary health care center in Pokhara.

**Methods:**

A descriptive cross-sectional study was conducted at Gandaki Medical College Teaching Hospital, Pokhara. After ethical approval from the institutional review board with Ref No: 070/2077/2078, endoscopic records of 889 patients undergoing upper gastrointestinal endoscopy from May 2018 to April 2019 were retrospectively reviewed. The convenience sampling method was used. Data entry and descriptive analysis were done in SPSS version 21.0. Descriptive statistics were performed.

**Results:**

A total of 889 patients undergoing upper gastrointestinal endoscopy during a period of 12 months period were studied. Among them, females were 472 (53.1%) and males were 417 (46.9%). The mean age of the study population was 45.6 years (SD, 16.86). The majority of the patients belonged to the age group 30 to 50 years. Gastritis was the most common finding in 452 (50.8%) cases, followed by gastroduodenal ulcer as the second commonest.

**Conclusions:**

Gastritis was the most common finding in the patients undergoing upper gastrointestinal endoscopy. A significant proportion of people had normal endoscopy findings. Optimal selection of cases is needed to avoid overuse in younger and encourage use in older populations.

## INTRODUCTION

Endoscopic examination is important in evaluating and managing gastrointestinal problems. Adverse events are inherent in the performance of UGI endoscopic procedures.^1^ American Society for Gastrointestinal Endoscopy recommends initial endoscopy for new-onset dyspepsia in patients 50 years of age of older or those with alarm features.^2^ Various indicators classified as preprocedure, intraprocedure, and post-procedure indicators are advised as quality indicators for endoscopic procedures.^3^

Upper gastrointestinal endoscopy is indicated for a number of diagnostic and therapeutic purposes, which commonly includes those with dyspeptic symptoms, cases of upper gastrointestinal bleed, removal of foreign bodies, selected cases of portal hypertension for the screening of varices.^4^ The most common findings include gastritis. Other findings include esophagitis, gastric ulcer, duodenal ulcer, biliary gastritis, gastric mass. Normal findings were also seen in varying percentages in different studies done.^5-8^

The objective of the study was to study the endoscopic findings in the patients undergoing upper gastrointestinal endoscopy in a tertiary care center.

## METHODS

A descriptive cross-sectional study was conducted among 889 patients attending the endoscopic department of Gandaki Medical College Teaching Hospital and Research Center, Pokhara, from May 1^st^ 2018 to April 1^st^ 2019.

Ethical clearance was obtained from the Institutional Review Board of GMCTH; Pokhara prior to data collection. (Ref No: 070/2077/2078)

All patients undergoing endoscopy during the study period were included and no exclusions were made. Hence, the whole sampling technique was used in the study. Sample size calculation was done using the formula


$$\begin{array}{ll} \text{n} & ={\text{Z}}^{\text{2}}\times \text{p}\times \text{q}/{\text{e}}^{\text{2}}\\ & =2.326^{2}\times 0.5\times 0.5/0.04^{\text{2}}\\ & =845 \end{array}$$

Where,

n = sample size
Z = 2.326 at 98% Confidence Interval (CI)
p = prevalence of elderly malnutrition i.e. 50%
q = 1-p
e = margin of error, 4%

Data was collected for a period of four weeks from 15^th^ August to 15^th^ September, 2020 from the record department after ethical approval. Data was collected using proforma and entered into IBM SPSS version 21.

Descriptive statistics such as frequency, percentage and mean were used. Confidentiality and privacy of the data were maintained by utilizing it just for the study purpose.

## RESULTS

Out of a total of 889 cases, 417 (46.9%) were males, 472 (53.1%) were females. Five hundred and one (56.7%) were of the age group of 25 to 54 years (Table 1).

**Table 1:** Age-wise distribution across male and female gender (n = 889).

Age-group/Gender	Male n (%)	Female n (%)	Total n (%)
≤24 yrs	47 (5.3)	54 (6.1)	101 (11.4)
25 to 34	73 (8.2)	89 (10)	162 (18.2)
35 to 44	80 (9)	87 (9.8)	167 (18.8)
45 to 54	71 (8)	101 (11.4)	172 (19.3)
55 to 64	66 (7.4)	83 (9.3)	149 (16.8)
65 and above	80 (9)	58 (6.5)	138 (15.5)
Total	417 (46.9)	472 (53.1)	

Among the total patients, approximately half (50.8%) of them had findings of gastritis. Among the total patients, 452 (50.8%) of them had findings of gastritis. About 175 (19.7%) had a gastroduodenal ulcer in endoscopy. Normal endoscopy was present in 130 patients (Table 2).

**Table 2:** 

G.I. endoscopic findings	n (%)
Gastritis	452 (50.8)
Gastroduodenal ulcer	175 (19.7)
Normal	130 (14.6)
Oesophageal varices	47 (5.3)
Esophagitis	40 (4.5)
Mass -Oesophageal/Gastric	21 (2.4)
Biliary gastropathy	10 (1.1)
Portal HTNsive gastropathy	8 (0.9)
Impacted bone	6 (0.7)
Total	889 (100.0)

Most common indication for undergoing upper G.I. endoscopy was abdominal pain (65.2%) followed by heartburn (18.8%). Sixty-five patients presented to the endoscopy unit due to gastrointestinal bleed. A few patients also went endoscopy as a part of the management of cirrhosis (Table 3).

**Table 3:** Indications behind upper GI endoscopy (n = 889).

Indications	n (%)
Abdominal Pain	580 (65.2)
Heartburn	167 (18.8)
G.I. Bleed	65 (7.3)
Cirrhosis	35 (3.9)
Dysphasia	12 (1.3)
Weight Loss	11 (1.2)
Anaemia	10 (1.1)
Vomiting	9 (1.0)

**Figure 1.**
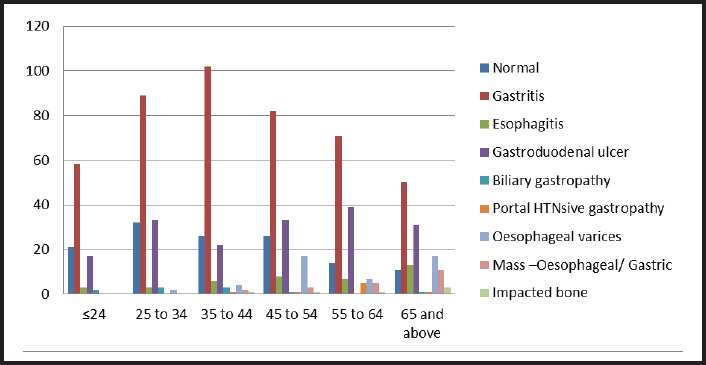
Upper Gastrointestinal Findings across Age-groups.

Further analysis of endoscopic findings across various age groups revealed the frequency of normal findings decreased with the increasing age group. There was an increased frequency of esophageal varices from 45 years onwards. The findings of gastric/esophageal mass were also higher in the older age group (Figure 1).

## DISCUSSION

Esophagogastroduodenoscopy (EGD) affords an excellent view of mucosal surfaces of the esophagus, stomach, and proximal duodenum. Diagnostic observations are made concerning focal benign or malignant lesions, diffuse mucosal changes, luminal obstruction, motility, and extrinsic compression by contiguous structures. Common therapeutic endoscopic procedures include polypectomy, dilation of strictures, stent placement, removal of foreign bodies, gastrostomy, G.I. bleeding treatment with injection, banding, coagulation, sclerotherapy, and endoscopic therapy of intestinal metaplasia.^4^

The study showed a dominance of females (53.1%) presenting to the endoscopic unit. This finding was similar to other studies done in Nepal in other provinces.^5,6^ Contrary to this, other studies were done in Nepal Medical college and Lumbini Medical College had slightly more males than females.^7^ A retrospective cohort study of patients who had upper gastrointestinal endoscopy for a period of 4 years in a rural hospital in Nigeria also showed male predominance (52.5%).^9^ The similarities and disparities are likely due to the differences in health-seeking behaviors of people in different provinces and nations, along with the different design studies.

Age-wise distribution showed that the majority were between ages 45 to 54 years, followed by younger age groups. This was similar to a study done in 2012 A.D in Kathmandu.^7^ In contrast, another study in 2014 A.D in Patan hospital had predominance in below 40 years age.^6^ The timing of the study may have an impact on the findings. This shows younger patients underwent endoscopic units frequently as compared to the older age group. The likely reason may be due to increased concern for the symptoms in the younger age group. Older patients may not have easy access to go to hospitals with the endoscopic facility.

Abdominal pain was the most common indication for undergoing endoscopy in this study. This was similar to other studies done in different years across different institutions and hospitals.^5,10^ Similar finding was also seen in studies done in India, Uganda, Ghana and Nigeria.^9,11-13^ Gastrointestinal bleeding was the presentation in few, though not the majority of the patients. Alarming symptoms like vomiting, weight loss and anemia were present in a negligible number of cases. Due to the study's retrospective nature, the patients' chief complaints may not have been well recorded and so seen in a negligible number only.

Findings on upper gastrointestinal endoscopy were variable. Gastritis was the most common finding accounting for about half of the cases. This was similar to other studies done in Nepal in last few years.^5,6,8,10^

Also, studies done outside Nepal showed gastritis as the commonest finding in patients undergoing endoscopy.^9,11,12,14,15^

The presence of gastroduodenal ulcer was also found in approximately one-fifth of the cases. This is higher than the percentage of patients presenting with symptoms of gastrointestinal bleed in this study. This shows that such patients may present with other non-specific symptoms also. Hence high suspicion should be looked for such cases and mainly those not relieved symptomatically with a trial of anti-gastric agents.

Normal findings on endoscopy were present in 15% of cases. This is similar to the study done by Obayo et al. in a teaching hospital in Uganda.^12^ In another study done by Patel et al. among patients with persistent upper abdominal pain, 22% of cases had normal upper gastrointestinal endoscopy findings.^11^ This is a significant number that may account for the overutilization of endoscopy in day-to-day practice. This aspect has also been highlighted in the study done by Bohara et al. in 2017 A.D in a medical college in Kathmandu.^10^ Contrary to this, a study was done by Joshi et al. in a government hospital showed only 2% of cases had normal endoscopic findings.^5^ The difference is likely due to a difference in the total number of cases as well as the difference in the setup in government and private medical colleges. Selection of cases as per guidelines, age and elucidating the presence of alarming symptoms may help to decrease normal cases of endoscopy.

Sub-group analysis as per the symptoms and age group and endoscopic diagnosis could further stratify the study. However, due to the study's retrospective nature, the details may not be complete missing the detailed symptoms, examination findings, lab findings, and other pertinent issues. In this study, the researcher didn't study H. pylori status, patients' personal habits, and the history of drug intake. Further prospective studies are needed to address these issues. A larger sample and multicentre study are needed for the generalization of the study.

## CONCLUSIONS

Gastritis was the most common finding. Normal findings in patients undergoing upper gastrointestinal endoscopy were seen in a significant number. Older age group people were of few numbers. Appropriate screening for indications in the younger age group is needed to avoid overuse of endoscopy in those age groups so as to minimize the possible complications. The older age group should be addressed and educated to seek endoscopy in needed cases or symptoms.
